# A novel SigB(Q225P) mutation in *Staphylococcus aureus* retains virulence but promotes biofilm formation

**DOI:** 10.1038/s41426-018-0078-1

**Published:** 2018-04-25

**Authors:** Hui Liu, Weilong Shang, Zhen Hu, Ying Zheng, Jizhen Yuan, Qiwen Hu, Huagang Peng, Xinyu Cai, Li Tan, Shu Li, Junmin Zhu, Ming Li, Xiaomei Hu, Renjie Zhou, Xiancai Rao, Yi Yang

**Affiliations:** 1 Department of Microbiology, College of Basic Medical Sciences, Third Military Medical University, 400038 Chongqing, China; 2 Department of Emergency, Xinqiao Hospital, Third Military Medical University, 400037 Chongqing, China

## Abstract

*Staphylococcus aureus* is an important pathogen that produces abundant virulence factors, which cause various diseases that burden human health worldwide. The stress response regulon called sigma factor B (SigB) is a well-characterized global regulator that is involved in the regulation of *S. aureus* virulence, pigmentation, and biofilm formation. However, the regulatory network upon SigB in *S. aureus* is incompletely described. Here, we identified a novel substitution mutation, SigB(Q225P), which contributed the nonpigmented phenotype of *S. aureus*. The *S. aureus* mutant carrying SigB(Q225P) substitution lacks staphyloxanthin, a key virulence factor in protecting bacteria from host-oxidant killing, but retains bacterial pathogenicity with pleiotropic alterations in virulence factors, resulting in similar lethality and abscess formation ability in animal models. We also reported the SigB(Q225P) promotion of biofilm formation in *S. aureus*. Real-time quantitative polymerase chain reaction (RT-qPCR) revealed that the expression of *nuc* gene, which encodes thermonuclease, was significantly downregulated, resulting in accumulation of eDNA in the biofilm of SigB(Q225P) mutant strain. LacZ reporter assay showed that SigB(Q225P) influenced the activity of *nuc* promoter. Furthermore, electrophoretic mobility shift assay (EMSA) and Bio-layer interferometry (BLI) assay revealed that both SigB and SigB(Q225P) proteins could directly bind to *nuc* gene promoter; however, the binding activity decreased for SigB(Q225P). Our data renewed the understanding of the relationship between *S. aureus* golden pigment and its virulence and suggested that a single substitution mutation in SigB might enhance the biofilm formation of *S. aureus* by directly downregulating *nuc* expression.

## Introduction

*Staphylococcus aureus* is a notorious pathogen capable of causing various diseases, ranging from acute skin and soft tissue infections to chronic or persistent endocarditis, osteomyelitis, and implant infections^1–3^. *S. aureus* infections have brought great economic and social burdens worldwide. In China, according to China Antimicrobial Resistance Surveillance System (http://www.carss.cn/), *S. aureus* ranked the first among Gram-positive pathogenic bacteria. In the United States, a single community-associated methicillin-resistant *S. aureus* imposes an annual burden of about $2 billion on third-party payers and $10 billion on society^4^. *S. aureus* bacteria frequently act as versatile members that develop surface-attached community-designed biofilms to protect themselves from antibiotics treatment, host-immune killing, and other unfavorable environments^5–8^ and to activate the production of virulence factors contributing to bacterial colonization, persistence, and spreading^9, 10^. Nevertheless, the regulatory network underlying biofilm formation of *S. aureus* is extremely complicated but poorly understood.

*S. aureus* biofilms consist of complex extracellular matrixes (ECMs), including polysaccharide intercellular adhesion (PIA), proteins, and extracellular DNA (eDNA)^11^. Alternative sigma factor B (SigB), which majorly modulates the stress responses of several Gram-positive bacteria^12^, plays essential roles in regulating biofilm formation, virulence expression, and pigment synthesis of *S. aureus*^13–17^. The biosynthesis of staphyloxanthin, a key virulence factor for protecting *S. aureus* from host-oxidant killing *in vivo*^18^, depends on the alternative SigB^19^. The increased expression of SigB is always accompanied by enhanced biofilm formation^20^, whereas *sigB* gene-deleted mutant is unable to create a biofilm in both *S. aureus*^21^ and *S. epidermidis*^22^. *S. aureus* variants carrying certain substitution mutation in SigB (such as L242P) or truncated SigB do not produce pigment nor form biofilm^23^.

Theoretically, one substitution mutation in a certain molecule has three modes of influence (increase, decrease, or no change) on certain phenotype as compared with the wild-type molecule. However, in transcriptional regulators such as SigB, mutations in the DNA-binding domain tend to attenuate the protein’s function. In this study, we obtained a nonpigmented variant (XQW) from Luria-Bertain (LB) agar plates cultivated in the fifth passage of *S. aureus* XQ, a highly virulent strain isolated from the pus of a 17-year-old adolescent^24^. Genome sequencing and comparison revealed that XQW carried a novel SigB mutation, SigB(Q225P). Allelic replacement experiments in *S. aureus* Newman indicated that SigB(Q225P) mutation was responsible for the nonpigmented phenotype of *S. aureus*. However, the lack of staphyloxanthin did not affect bacterial virulence in animal killing and skin abscess formation in mouse models. SigB(Q225P) enhanced the biofilm formation of *S. aureus*. Further study showed that the increased biofilm of XQW was associated with the decreased *nuc* (encoding thermonuclease) expression of bacteria, resulting in accumulation of eDNA in the biofilm ECMs. LacZ reporter assay, electrophoretic mobility shift assay (EMSA), and Bio-layer interferometry (BLI) assay showed that SigB(Q225P) mutation weakened the direct interaction between SigB protein and *nuc* gene promoter, which consequently reduced *nuc* expression and enhanced biofilm formation. Our study provided initial evidence that a substitution mutation of SigB(Q225P) could increase the biofilm formation of *S. aureus*. The underlying mechanism has also been elucidated.

## Results

### SigB(Q225P) mutation contributes to the nonpigmented phenotypes of *S. aureus*

**Fig. 1:**
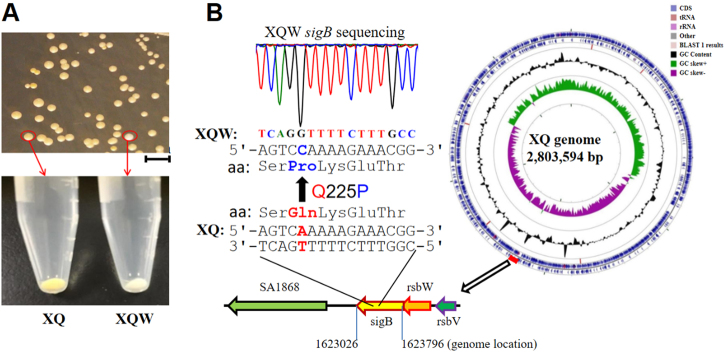
Nonpigmented phenotypes of *S. aureus* XQW and the SigB(Q225P) mutation. **a** XQ strain was cultured with LB medium, and the white colonies were observed on an LB plate. The nonpigmented phenotype was stable in the common TSB medium and was termed XQW strain as indicated. **b** Genome comparison revealed the SigB(Q225P) mutation in XQW. XQ contained the wild-type *sigB* gene, located approximately at 7 o’clock of its circular genome, whereas XQW contained a missense mutation in *sigB*(A674C), corresponding to a Q225P substitution in SigB factor, which was further confirmed by PCR amplification and DNA sequencing

The genetic plasticity enables *S. aureus*, a versatile microorganism, to adapt to the environment^23^. We previously isolated a highly virulent ST121/*agr*-IV methicillin-susceptible *S. aureus* (MSSA, termed XQ strain)^24^. After five passages of XQ in laboratory with LB medium, about 2% colonies on the LB agar plate exhibited white phenotypes (Fig. 1a). One of the colorless colonies was then inoculated into tryptic soy broth (TSB), and its white phenotype was stable and named XQW strain (Fig. 1a).

The biosynthesis of golden carotenoid pigment (staphyloxanthin) in *S. aureus* is controlled by the *crtOPQMN* operon. Many other regulators, such as SigB, IspA, CitZ, CitB, and CcpE, and small RNA SsrA, influence the production of *S. aureus* staphyloxanthin^13, 18^. Genome sequencing of XQ (GenBank accession number: CP013137.1) and XQW was conducted to identify the gene responsible for the white phenotype of XQW. Genome comparison revealed 527 single-nucleotide polymorphisms (SNPs) in XQW genome; 392 of which were involved in 161 genes, which altered the corresponding amino acids (Supplementary Table S1). XQW contained a missense mutation in *sigB*(A674C), which encoded glutamine/proline substitution Q225P (Fig. 1b). This mutation was located in the putative DNA-binding domain of SigB that spans residues of 208–247;^25^ this mutated gene may be responsible for the white phenotype of XQW.

**Fig. 2:**
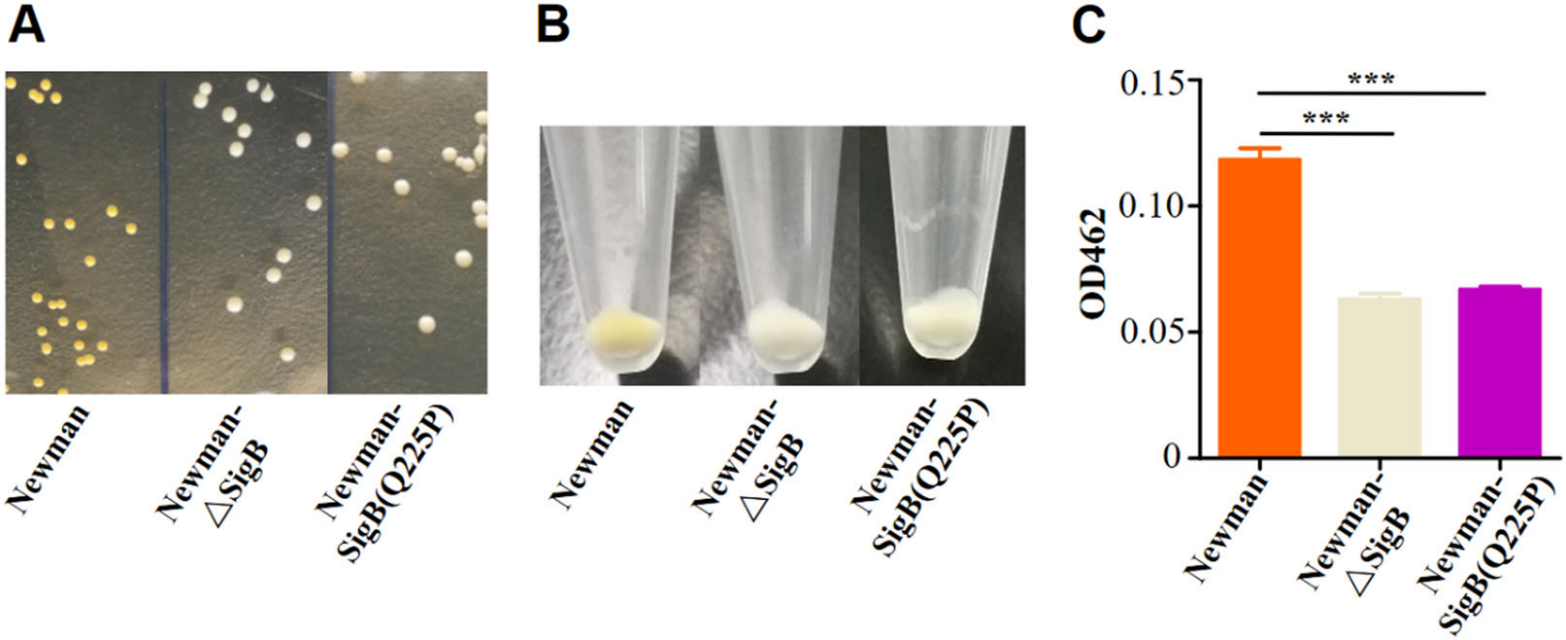
*S. aureus* Newman with SigB(Q225P) mutation exhibited the nonpigmented phenotype. **a** Colonies of Newman, Newman-ΔSigB, and Newman-SigB(Q225P) on TSB plates. **b** Cell pellets of Newman, Newman-ΔSigB, and Newman-SigB(Q225P) from TSB cultures. **c** Carotenoids produced by Newman, Newman-ΔSigB, and Newman-SigB(Q225P) were extracted, and the OD462 value was determined (*n* = 3). ***P *<* 0.001

Due to the difficulty of genetic manipulation of the clinical isolate XQ, we introduced the SigB(Q225P) mutation into the strain Newman, a well-characterized MSSA strain, and tested the relationship between the mutation and the white colony phenotype. As expected, Newman-SigB(Q225P) and SigB mutant strain Newman-ΔSigB revealed nonpigmented phenotypes similar to those of XQW (Fig. 2a, b). The production of carotenoids was also determined, and the results indicated that Newman-SigB(Q225P) synthesized a decreased level of carotenoids as compared with wild-type Newman (Fig. 2c). These data suggested that the SigB(Q225P) mutation in *S. aureus* contributed to the nonpigmented phenotypes of *S. aureus* variants.

### SigB(Q225P) mutation retains the virulence of *S. aureus*

**Fig. 3:**
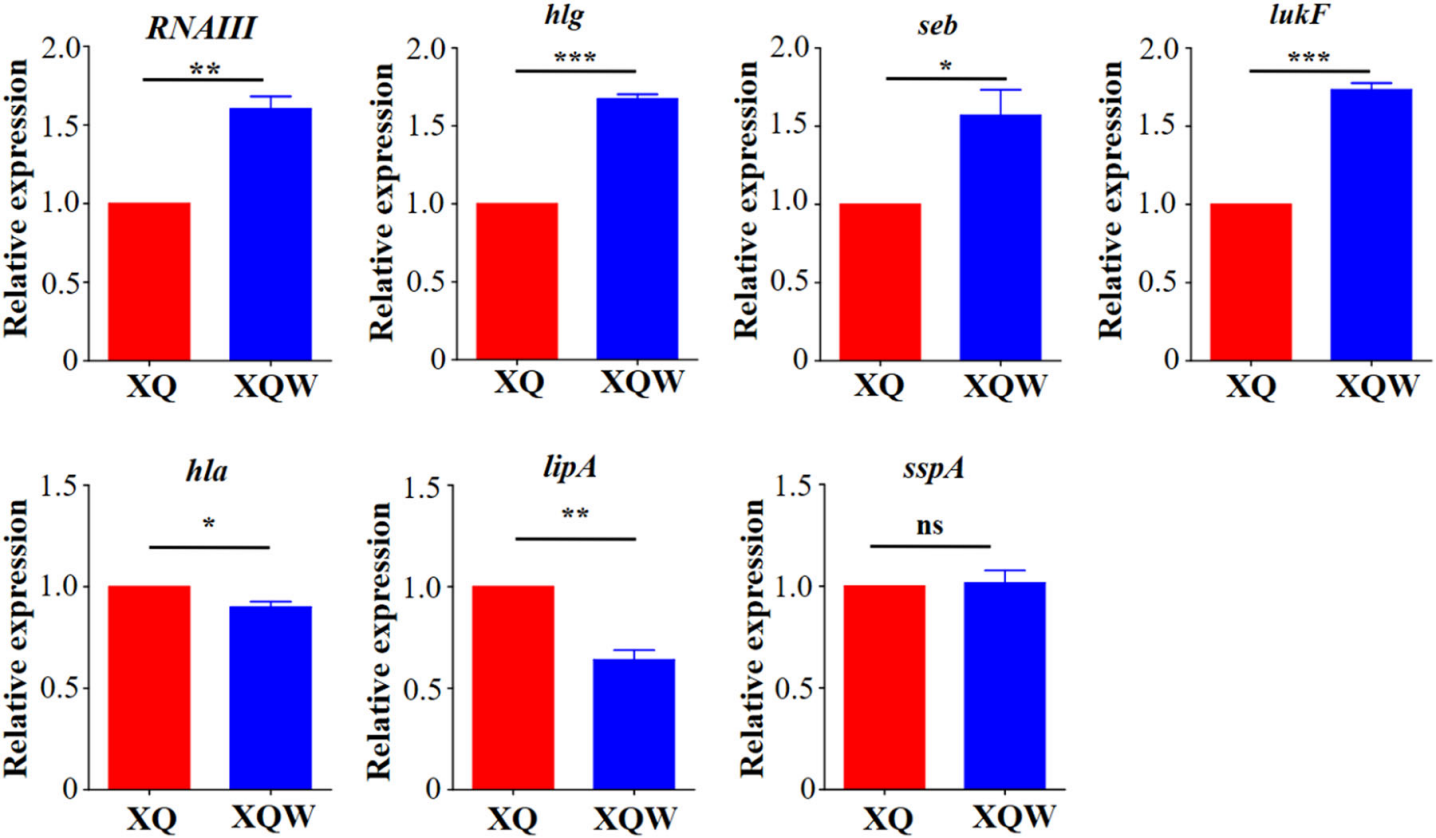
Expression levels of virulence genes (*RNAIII*, *hlg*, *seb*, *lukF, hla*, *lipA*, and *sspA*) in XQ and XQW determined by RT-qPCR. Expression of each gene of interest in XQ strain was normalized to the 16S RNA gene expression and adjusted to 1.0, and its relative expression in XQW strain was indicated. *P *<* 0.05, **P *<* 0.01, ***P *<* 0.001, and ns represents no significance, *n* = 3

Staphyloxanthin is an important virulence factor to protect *S. aureus* from host-oxidant killing^26^. Inhibitors targeting staphyloxanthin biosynthesis impair *S. aureus* virulence^18, 27^. The expression levels of several major virulence factors were detected by RT-qPCR to investigate the influence of SigB(Q225P) mutation on the virulence of XQW (Fig. 3). The results revealed that *RNAIII*, which controls the expression of many virulence factors^28^, was significantly upregulated in XQW as compared with that in XQ. The genes encoding γ-hemolysin (*hlg*), staphylococcal enterotoxin B (*seb*), and Pantone-Valentine leucocidin (*lukF*) were also upregulated, whereas those encoding α-hemolysin (*hla*) and lipase (*lipA*) were significantly downregulated in XQW. No alteration in the expression of gene encoding V8 serine protease (*sspA*) was observed between XQW and XQ.

**Fig. 4:**
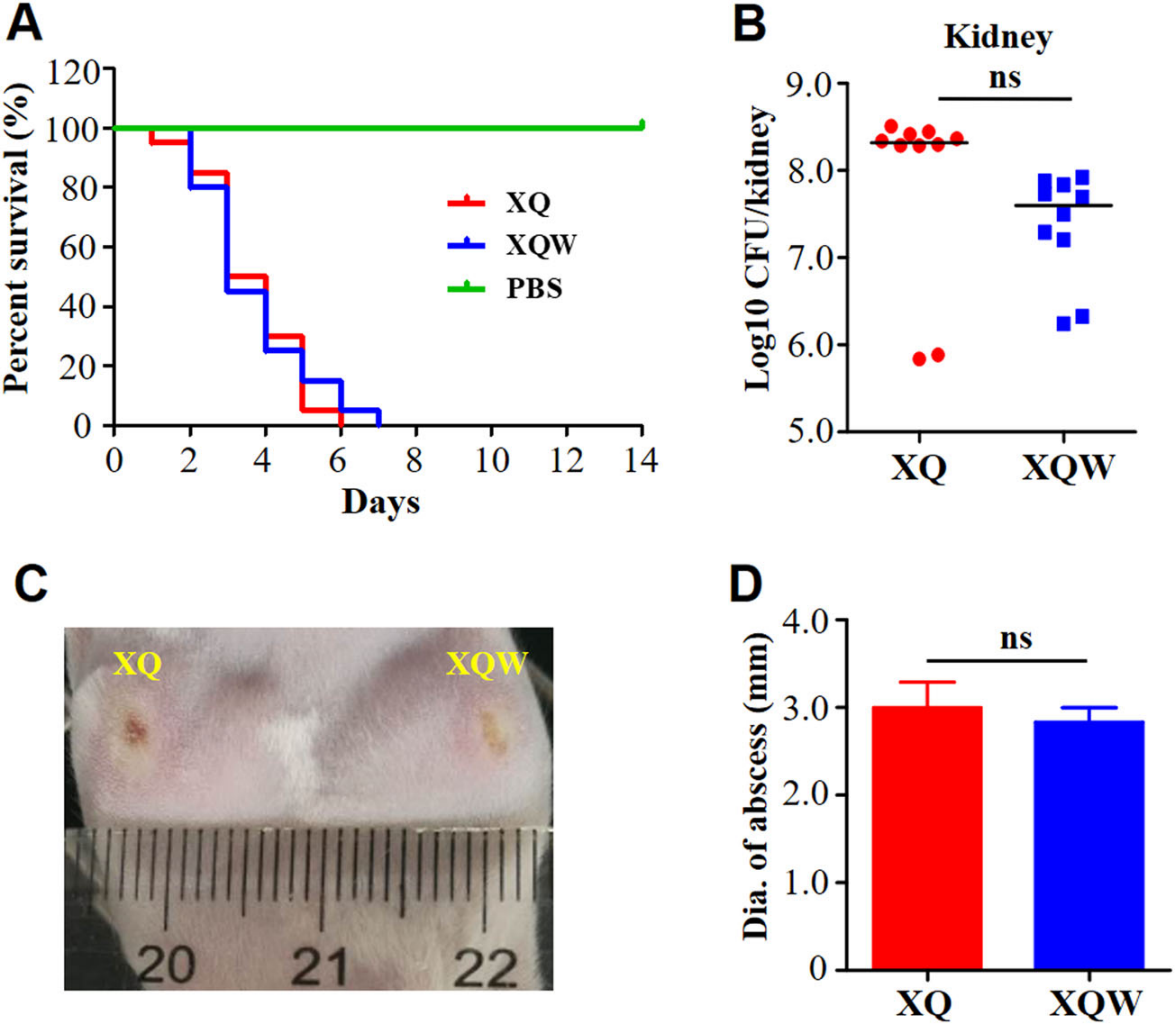
Virulence of XQ and XQW in mouse models. **a** Survival analysis. Mice were injected through tail vein with 4 × 10^7^ CFU of XQ, XQW, or PBS, and the survival rates were calculated. Number of mice used: *n* *=* 10. **b** Bacterial loads. Mice were injected through tail vein with 1 × 10^7^ CFU of XQ or XQW; bacterial load in the kidney tissue was counted four days post-injection. **c** Skin abscess formation. The hairs on the back of the mice were removed using 6% Na_2_S, then mice were respectively injected with XQ and XQW on each side. The skin abscesses were photographed 10 days after injection. **d** Diameter of abscess area was measured and represented as mean ± SD (*n* ≥ 3); ns represents no significance

Although the alteration of virulence factor expression in XQW was pleiotropic (Fig. 3), the overall lethality was similar in mice (*n* *=* 10) challenged by tail vein injection with 4 × 10^7^ CFU of XQ or XQW bacteria (Fig. 4a). The bacterial loads were also not significantly changed in the kidney (Fig. 4b), liver (Supplementary Figure S1A), and spleen (Supplementary Figure S1B) of infected mice. As a highly virulent invasive clinical isolate, XQ and XQW caused similar abscess in a skin-infected model (Fig. 4c, d). Moreover, the pathogenicity of Newman and Newman-SigB(Q225P) was also similarly evaluated by survival rate, bacterial loads, and abscess formation abilities (Supplementary Figure S2). These data indicated that the SigB(Q225P) mutation in *S. aureus* impaired the staphyloxanthin production but retained bacterial virulence.

### SigB(Q225P) mutation promotes the biofilm formation of *S. aureus*

**Fig. 5:**
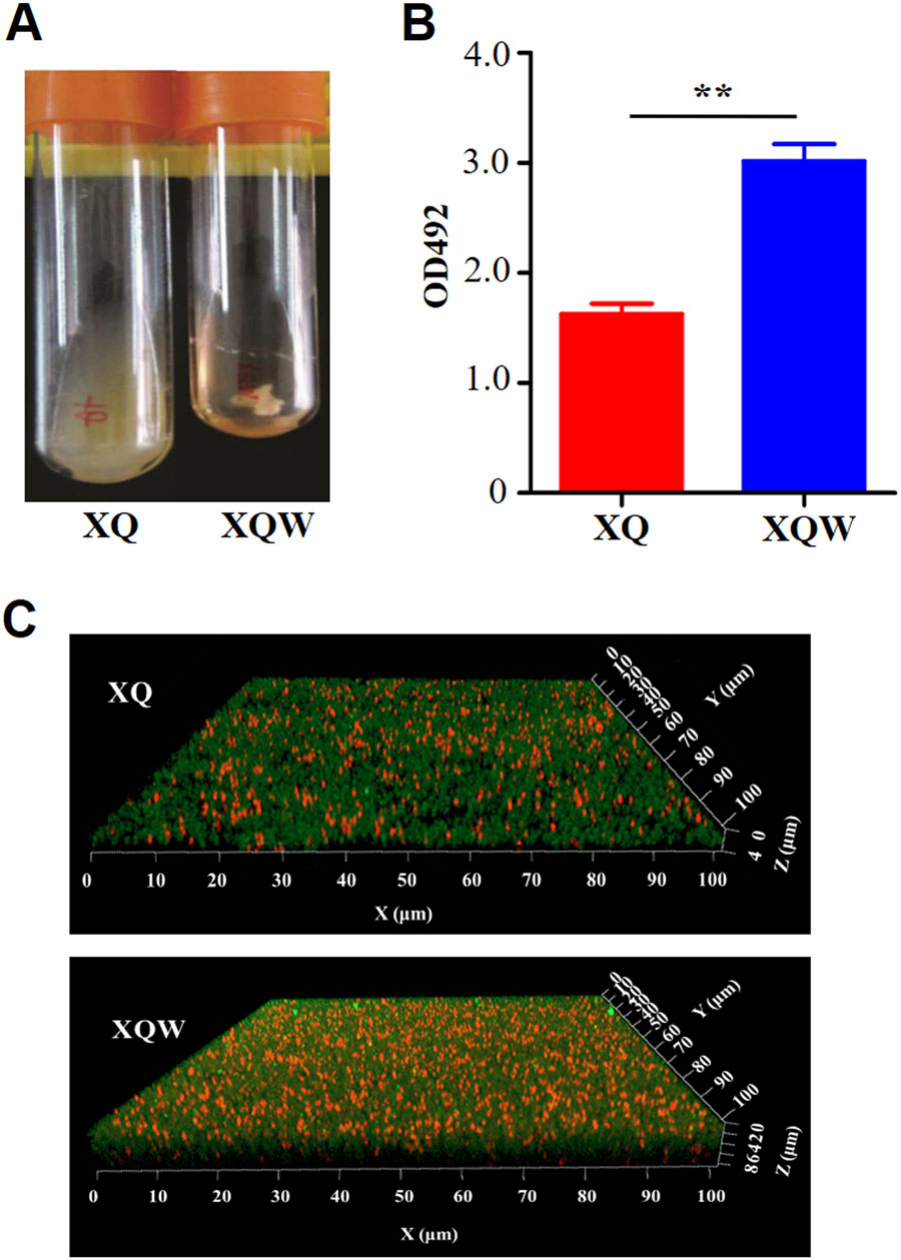
Detection of biofilm formation in *S. aureus* XQ and XQW. **a** XQW strain exhibited flocculent growth in LB medium. **b** Detection of biofilm formation with crystal violet staining method. The OD492 values were represented as mean ± SD (*n* = 3). **P *<* 0.01. **c** Biofilms derived from XQ and XQW strains were observed under a confocal scanning laser microscope. The polysaccharides in biofilm were stained green, and the bacteria were stained red. XQW produced thicker and more compact biofilm than XQ

The growth rate of XQW was slightly faster than that of XQ (Supplementary Figure S3A); while Newman-SigB(Q225P) showed similar growth rate to that of the wild-type Newman (Supplementary Figure S3B). Interestingly, XQW exhibited flocculent growth in the LB medium, whereas XQ maintained turbid growth (Fig. 5a). SigB positively regulated the biofilm formation in *S. aureus*; SigB mutant (ΔSigB) or *S. aureus* with a substitution mutation in SigB (such as L242P) was unable to develop a biofilm^21, 23^. According to the flocculent growth phenomenon, we speculated that SigB(Q225P) mutation in XQW promoted its biofilm formation. Crystal violet staining method was used to detect the biofilm formation of XQ and XQW in a 24-well plate and thus confirm this conjecture. The results revealed that XQW had stronger biofilm formation ability than XQ (Fig. 5b). Confocal scanning laser microscopy also showed that XQW produced thicker and more compact biofilm than XQ (Fig. 5c), indicating that the biofilm formation in XQW was significantly enhanced.

**Fig. 6:**
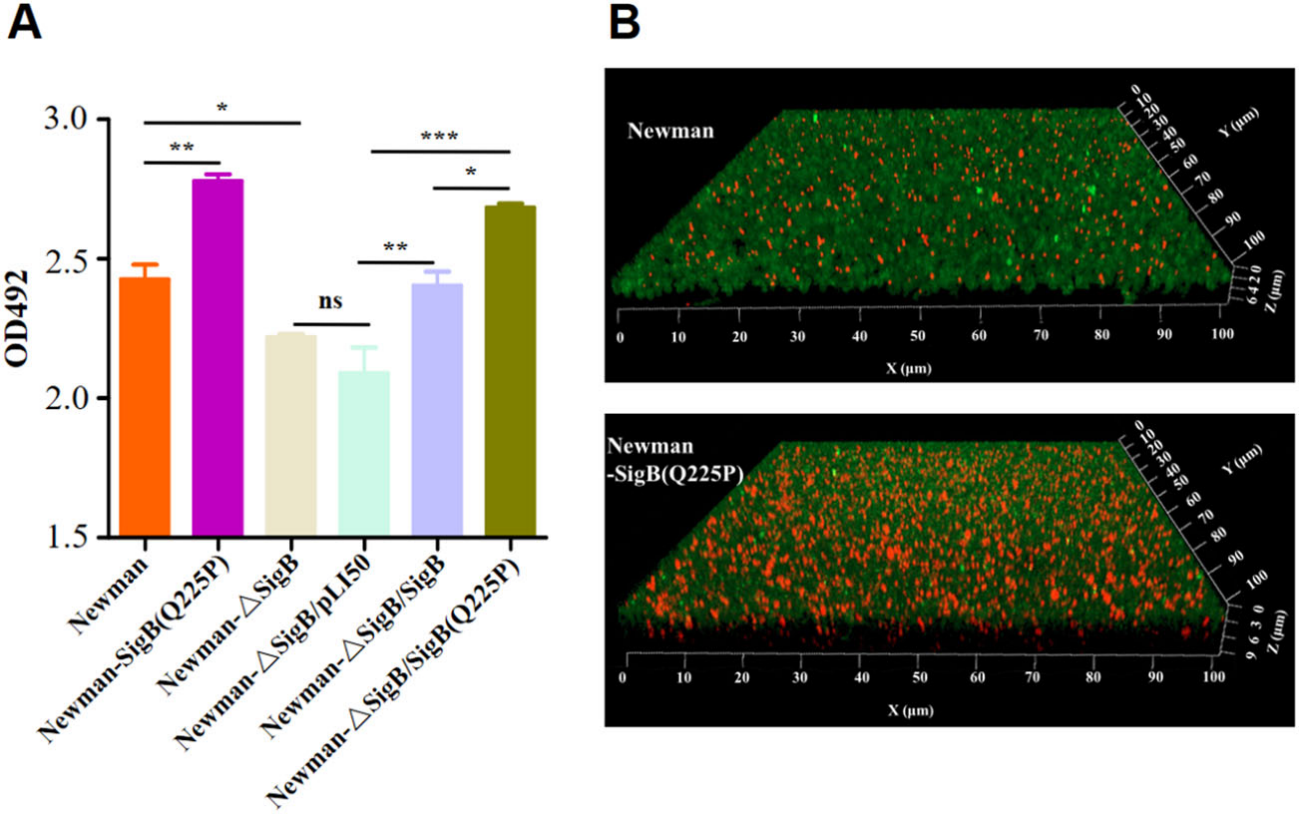
Detection of biofilm formation in *S. aureus* strain Newman and its derivatives. **a** Crystal violet staining method. Biofilms derived from Newman and its derivatives were stained with crystal violet, and the OD492 was detected (*n* *=* 3). *P *<* 0.05, **P *<* 0.01, ***P *<* 0.001, and ns represents no significance. **b** Observation of biofilm under a confocal microscope. Biofilms produced by Newman and Newman-SigB(Q225P) were indicated. The polysaccharides in biofilm were stained green, and the bacteria were stained red. Newman-SigB(Q225P) exhibited stronger biofilm developing ability than Newman

By genetically manipulating *S. aureus* strain Newman and its derivatives, crystal violet staining revealed that the biofilm formation of Newman-SigB(Q225P) was significantly increased compared with that of Newman (Fig. 6a). This phenomenon was further confirmed by observing biofilms under a confocal microscope (Fig. 6b). However, the biofilm formation of Newman-ΔSigB was decreased when compared with that of Newman. The supplement of the wild-type SigB to Newman-ΔSigB (Newman-ΔSigB/SigB) elevated its biofilm formation similarly to wild-type Newman. Newman-ΔSigB/SigB increased its biofilm formation compared with Newman-ΔSigB carrying an empty plasmid (Newman-ΔSigB/pLI50). Moreover, supplement of the SigB(Q225P) to Newman-ΔSigB (Newman-ΔSigB/SigB(Q225P)) even enhanced the biofilm formation compared with both Newman-ΔSigB and Newman-ΔSigB/pLI50 (Fig. 6a). These data indicated that SigB(Q225P) is the key mutation for promoting biofilm formation in *S. aureus*.

### SigB(Q225P) promotes biofilm formation of *S. aureus* by direct downregulation of *nuc* expression

**Fig. 7:**
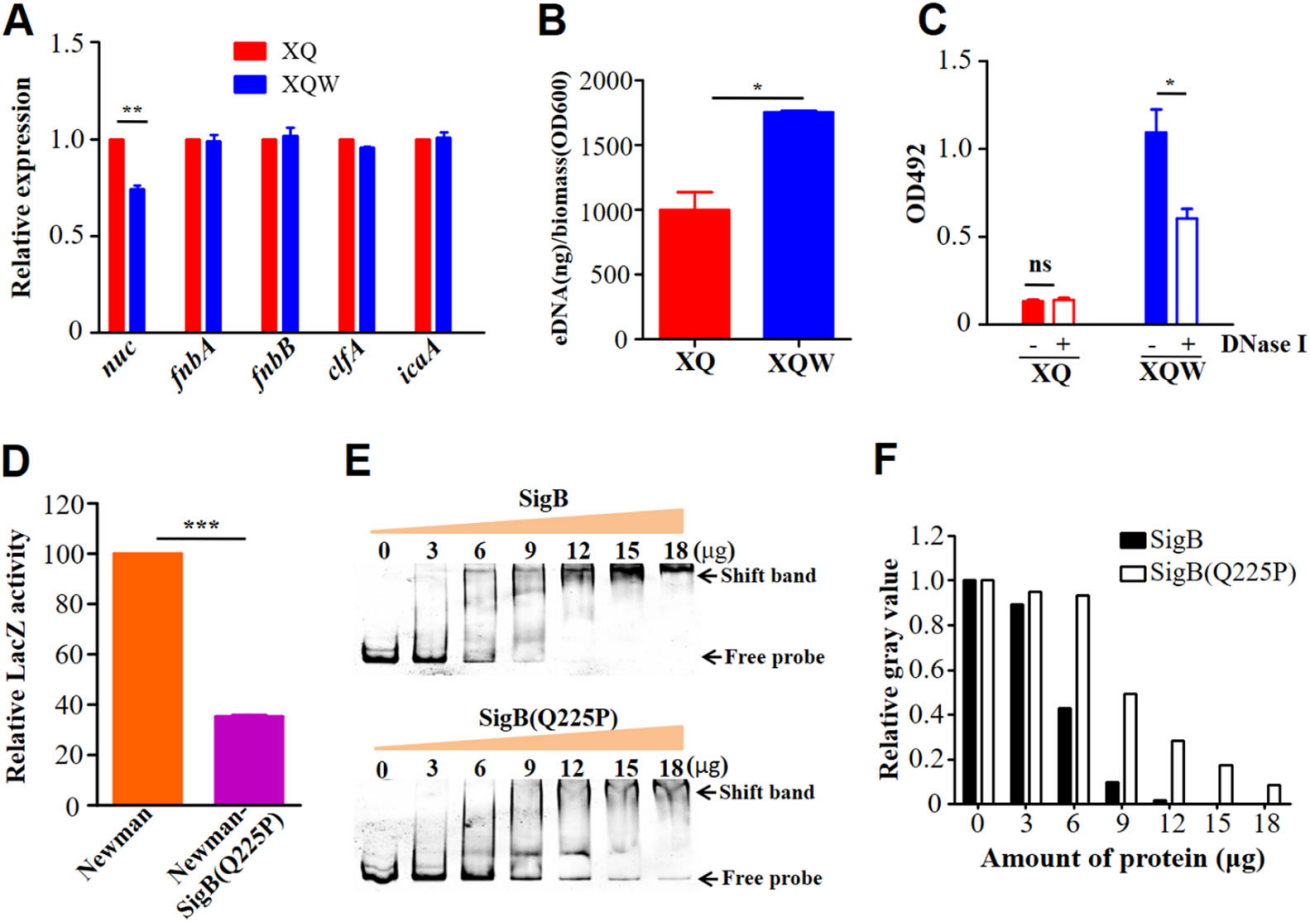
SigB(Q225P) mutation promotes biofilm formation of *S. aureus* via direct downregulation of *nuc* expression. **a** RT-qPCR detection of the expression level of *nuc*, *fnbA*, *fnbB*, *clfA*, and *icaA* genes in XQ and XQW. Only the *nuc* expression was reduced in XQW strain. **P *<* 0.01. **b** eDNA was extracted and determined. XQW biofilms contained more eDNA than XQ. *P *<* 0.05. **c** DNase I treatment experiment. The biofilm formation in XQW was significantly reduced after co-incubation with DNase I compared with Tris buffer (20 mM, pH 7.5), whereas biofilm formation in XQ was not changed. *P *<* 0.05. **d** β-galactosidase assay. The pOS1-*nuc* promoter-*LacZ* reporter plasmid was transformed into Newman and Newman-SigB(Q225P), respectively. The LacZ activity was detected and represented as mean ± SD (*n* = 3). ***P *<* 0.001. **e** EMSA. Interaction between *nuc* gene promoter and SigB or SigB(Q225P) proteins was detected. The amount of free DNA probes and the shift bands were arrow-indicated. **f** Evaluation of gray value of the free probe in each lane using ImageJ software. The value of free probe in first lane (0 μg protein) was adjusted to 1.0, and the relative gray values in other lanes were calculated and indicated

One of the hallmark events in biofilm formation is the production of slime PIA controlled by the *icaADBC* operon^29^. However, *S. aureus* can develop an alternative *ica*-independent biofilm^30^, where the surface proteins, such as FnBPA/B, ClfA/B, Bap, SPA, and SasG^17, 31^, and extracellular DNA (eDNA) play important roles^21, 30^. The genes involved in the production of PIA, surface proteins, or eDNA were selected and tested by RT-qPCR to explore the mechanisms by which SigB(Q225P) promotes the biofilm formation in *S. aureus*. As shown in Fig. 7a, the *nuc* gene expression was significantly reduced in XQW as compared with that in XQ. The decreased *nuc* expression could also be observed in Newman-SigB(Q225P) as compared with Newman wild-type strain (Supplementary Figure S4). However, *nuc* expression was significantly upregulated in the *sigB*-deleted mutant Newman-ΔSigB, which is in accordance with those of a previous study^21^.

The thermonuclease encoded by *nuc* gene negatively regulates eDNA in *S. aureus* biofilm^32^. The reduced expression of *nuc* in XQW would lead to massive accumulation of eDNA in its biofilm development. The amount of eDNA in the biofilm was extracted and determined as described to confirm this hypothesis^33^. The results showed higher eDNA amount from XQW-derived biofilms than from XQ biofilms (Fig. 7b and Supplementary Figure S5A). In addition, when DNase I was added into the culture to compensate the decreased expression of thermonuclease, XQW-produced biofilm was significantly decreased, whereas biofilm formation of XQ was not affected (Fig. 7c and Supplementary Figure S5B). Our data suggested that the decreased *nuc* expression results in the accumulation of eDNA and may be associated with the enhanced biofilm formation in XQW.

**Fig. 8:**
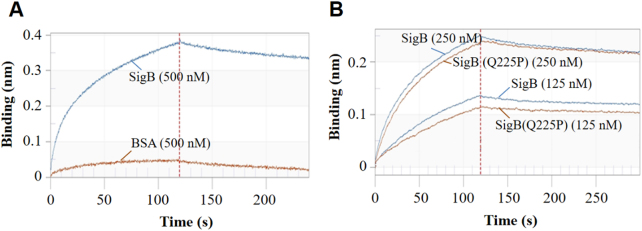
The binding sensorgrams (nM) for *nuc* promotor-SigB or SigB(Q225P) interaction using single cycle kinetic assay format. **a** The specificity of the system. BSA served as negative control. **b** SigB and SigB(Q225P) proteins interacted with *nuc* promotor in a dose-dependent manner

In *S. aureus* strain COL, SigB deletion led the upregulation of thermonuclease precursor^34^. A *nuc* promoter-controlled LacZ reporter plasmid was constructed and transformed into Newman and Newman-SigB(Q225P) strains to test the effect of SigB(Q225P) mutation on the *nuc* expression. LacZ assay revealed that the activity of *nuc* promoter in Newman-SigB(Q225P) was only 35.4% of that in the wild-type Newman (Fig. 7d), indicating that SigB(Q225P) mutation could decrease the expression of *nuc* gene. We prepared the recombinant SigB and SigB(Q225P) proteins (Supplementary Figure S6A), then used EMSA to determine whether SigB(Q225P) could directly regulate *nuc* gene or not. The results showed that both recombinant SigB and SigB(Q225P) proteins could bind to *nuc* gene promoter in a dose-dependent manner (Fig. 7e) but not to an irrelevant DNA fragment (Supplementary Figure S6B). Moreover, SigB(Q225P) protein exhibited decreased binding ability compared with the wild-type SigB (Fig. 7f). The band of free probe nearly disappeared when the amount of SigB protein was increased to 12 μg (Fig. 7e, top panel), whereas 18 μg of SigB(Q225P) could not completely exhaust the free probe (Fig. 7e, bottom panel). A BLI assay was also performed to quantitatively detect the DNA binding ability of SigB and SigB(Q225P) proteins. As shown in Fig. 8, 500 nM recombinant SigB could bind well to the *nuc* promotor DNA coated biosensor, whereas the same amount of bovine serum albumin (BSA, negative control) could not (Fig. 8a). SigB showed stronger *nuc* promotor binding ability (*k*_a_ = 1.48 × 10^4^, *k*_d_ = 1.67 × 10^−3^, *K*_D_ = 1.13 × 10^−7^) than SigB(Q225P) (*k*_a_  = 5.63 × 10^3^, *k*_d_ = 1.54 × 10^−3^, *K*_D_ = 2.73 × 10^−7^). These data indicated that SigB(Q225P) promoted biofilm formation in *S. aureus* by directly downregulating *nuc* expression, which results in eDNA accumulation.

## Discussion

Bacterial biofilms are finely organized structures, in which bacteria only constitute ~15% of biofilm volume and are also embedded in ECMs^17^. The production of ECMs depends on media composition and functional alterations of regulatory factors controlling biofilm formation^17, 35^. Although ECMs of *S. aureus* biofilms are mainly constituted by PIA, proteins, and eDNA, the regulatory network involved in biofilm formation is complicated. Extracellular proteases (Aur, SpL, SspA, and SspB)^20, 36–38^, thermonuclease (Nuc)^32, 39^, autolysins (LytM, LytN, and AtlA)^35^, toxins (PSMα, PSMβ, and δ-hemolysin)^40^, holin/antiholin molecules (CidA and LrgAB)^33^, two-component systems (Agr, WalKR, LytSR, SaeRS, ArlRS, and SrrAB)^17, 41–44^, global regulators (SigB, SarA, MgrA, and Rot)^16, 23, 29, 30, 34, 45, 46^, and non-coding RNAs (msaABCR)^35^ directly and/or indirectly regulate *S. aureus* biofilm formation. The alternative SigB is an essential regulator of *S. aureus* biofilm formation; deletion of *sigB* deprives biofilm formation in *S. aureus*^20, 21^. Savage et al^23^. reported that a single substitution mutation in SigB, L242P, failed to form a biofilm in *S. aureus*. However, in the present study, we identified a SigB(Q225P) mutation that could unexpectedly promote biofilm formation of *S. aureus*, at least in strain backgrounds of XQ and Newman. In *S. aureus* strain COL, Pane-Farre et al^34^. found that 13 genes are negatively regulated by SigB under alkaline stress conditions, including *nuc* that encodes thermonuclease precursor. However, our screen with RT-qPCR revealed that *nuc* was downregulated in XQW (Fig. 7a) and Newman-SigB(Q225P) (Supplementary Figure S4). LacZ reporter assay showed that SigB(Q225P) reduced *nuc* expression (Fig. 7d), and EMSA and BLI revealed that SigB binded to the *nuc* promoter directly, and the Q225P mutation lowered the DNA binding of SigB(Q225P) to the *nuc* promoter (Fig. 7e,f and 8b). *S. aureus* SigB is known to be a negative regulator of the *nuc* expression^47^, and the lower DNA binding of SigB(Q225P) downregulation of the *nuc* expression is puzzling. We speculate that SigB(Q225P) mutation may also downregulate SigB-controlled downstream regulatory factors, such as SpoVG, which can significantly promote the *nuc* expression by counteracting the repressing effect of SigB^47^. Therefore, the upregulation of the *nuc* expression by SigB(Q225P) mutation is presumably over-compensated by the lower level of SpoVG-related downregulation of the *nuc* expression. As a result, a phenotype of SigB(Q225P)-associated downregulation of *nuc* expression was observed in this study. However, SigB(Q225P) downregulates SpoVG and the later fine-tunes the nuc expression need further investigation.

The first characteristic in identifying XQW is its nonpigmented phenotype on the LB plate. As an important virulence factor, staphyloxanthin can impair neutrophil killing and protect bacteria against host innate immune system^26, 27^. Inhibiting the production of staphyloxanthin by small-molecule could attenuate *S. aureus* virulence^18^. We found that the nonpigmented XQW, which carries SigB(Q225P) mutation, retained its virulence similarly to its wild-type XQ in the overall mouse lethality, organ colonization, and skin abscess formation (Fig. 4). The virulence between isogenic Newman and Newman-SigB(Q225P) was not statistically different either (Supplementary Figure S2). This phenomenon might be reasonable because the virulence of a certain bacterial strain is the aggregate performance of its virulence factor profiles. Given the pathogenic role of a certain virulence factor, such as staphyloxanthin^26, 48^, the presence and absence of the factor might significantly influence the pathogenicity of the bacterial strain; however, when testing the pathogenic role of a regulator, especially the global regulator (such as SigB or SarA), a single substitution mutation of the regulator might not affect the overall pathogenicity due to the complicated alteration of virulence factor profiles. In the present study, we observed the pleiotropic changes of virulence factors in XQW that carried SigB(Q225P) mutation; the results indicated upregulation in *RNAIII*, *seb*, *hlg*, and *lukF* expression levels, downregulation in *hla* and *lipA* expression levels, and no changes in *sspA* (Fig. 3). The upregulation of *RNAIII* expression in *sigB* deleted strain was also reported in a previous study, where the *S. aureus* strain RN2482 was used^49^. The Q225P mutation located in the region 4 domain (residues 208–247) of SigB^23^, which is in charge the promoter-binding activity, and the virulence factor profiles could be site- and strain-specific due to SigB(Q225P) mutation. *S. aureus* produces many virulence factors involved in the pathogenicity of a certain strain, and its expression profile of virulence factors could be important in deciding the infectious outcome.

In conclusion, SigB is well established as an important transcriptional regulator in *S. aureus* to overcome stress conditions. Studies on the effects of substitution mutation on the SigB function were limited. We identified a SigB(Q225P) mutation that contributes to the nonpigmented phenotype of *S. aureus*. Although this mutation impairs staphyloxanthin production, *S. aureus* carrying SigB(Q225P) retains its pathogenicity with pleiotropic alterations in the virulence factors, at least in the strain background of XQ and Newman. Moreover, we revealed that the SigB(Q225P) mutation promotes the biofilm formation of *S. aureus* mainly through the direct downregulation of *nuc* expression. Our data indicated the important role of eDNA in the *S. aureus* biofilm formation and an exact pathway (SigB-Nuc-eDNA) controlling the amount of eDNA in ECMs of *S. aureus* biofilm.

## Materials and methods

### Bacterial strains, plasmids, and animals

*S. aureus* strain XQ was isolated from a patient with sepsis from our affiliated hospital^24^. The corresponding mutant XQW strain was obtained in the laboratory by serial passages of XQ in LB medium and the fifth passage plating of culture on an agar plate. *S. aureus* Newman was kindly provided by Professor Yu Lu (Jilin University, China). *Escherichia coli* DH5α was kept in our laboratory and used for plasmid construction.

Plasmids pBT2 and pLI50, which were used for gene mutation and complementation, respectively, were kindly provided by Professor Baolin Sun (University of Science and Technology of China, China), pET22b for protein preparation and pOS1-*LacZ* for gene promoter activity detection were kept in our laboratory.

Female 6–8 weeks old BALB/c mice were purchased from our University Animal Center. All animal experiments were approved by the local ethics board at the Third Military Medical University. Guidelines were established by Microbial Engineering under the Educational Committee on Animal Resources approved protocols for animal use.

### Genome sequencing and analysis

The genomes of XQ and XQW were prepared by using a TIANamp Bacteria DNA Kit (TIANGEN). The sequencing was performed by Department of Bioinformatics (Beijing Institute of Microbiology and Epidemiology), with an Ion Torrent PGM sequenator. The acquired contigs were assembled by Roche 454 Newbler 2.5 assembler while the gaps within the unaccomplished genome were filled by GapFiller (version 1.11). The accession number for XQ was obtained (CP013137.1) after submission of the whole-genome sequence to the GenBank. Genome comparison between XQ and XQW was carried out using Mauve software.

### Mutant construction

Newman is an ideal genetic manipulation *S. aureus* strain^50^. The SigB(Q225P) mutation was introduced to Newman to generate Newman-SigB(Q225P) by allelic replacement strategy as described^51^. To further illustrate the role of SigB(Q225P) on biofilm formation, a *sigB-*deleted mutant (Newman-ΔSigB) was also constructed. Newman-ΔSigB/SigB and Newman-ΔSigB/SigB(Q225P) were constructed with recombinant pLI50 plasmids for complement of the wild-type SigB and SigB(Q225P), respectively. The empty pLI50 plasmid transformed Newman-ΔSigB (Newman-ΔSigB/pLI50) served as negative control.

### Detection of carotenoids in *S. aureus*

*S. aureus* strains were cultured overnight at 37 °C. One milliliter of cell culture was collected and centrifuged. Cell pellets were washed thrice with sterilized water and then resuspended with 200 μL of methanol and heated for 3 min at 55 °C. After centrifuged at 10,000×*g* for 1 min, the supernatant was transferred to a 96-well plate, and the OD462 value was read using a Bio-Tek microplate reader. All experiments were repeated three times.

### Biofilm formation and detection

Biofilm formation was performed according to previous studies with some modifications^30^. Briefly, the overnight bacterial culture was 1:40 diluted in TSB medium containing 1% glucose and 2% NaCl. Two hundred microliters of the cell suspension were added to a sterile 96-well plate in triplicate. After incubation 24 h at 37 °C, wells were washed thrice with sterilized water, stained with 0.1% crystal violet for 1 min, and washed again with water. After that, the crystal violet was solubilized with 30% glacial acetic acid for 15 min. The relative biofilm formation was determined by detecting the OD492 values using a Bio-Tek microplate reader.

For confocal microscopy, *S. aureus* strain of interest was cultured on a clean coverslip put in a sterile six-well plate (5 mL per well). After incubation 24 h at 37 °C, the coverslip was fixed with 2.5% glutaraldehyde, washed with PBS, and stained with 50 mg/L of FITC-labeled ConA (Sigma) for 30 min. After being washed with PBS again, the coverslip was blocked with propidium iodide (5 mg/L, Sigma) for 8 min and observed under a confocal laser microscope (Leica TCS-NT). The polysaccharides of biofilm were stained green and the bacterial nucleoid was stained red.

### DNase I treatment assay

The overnight bacterial culture was 1:40 diluted in TSB medium containing 1% glucose, 2% NaCl, and 1 unit of DNase I, and cultured for 24 h in a 96-well plate. Wells were washed thrice with water, stained with crystal violet, and detected as described before.

### Extraction and quantification of eDNA

eDNA extraction and quantification was performed according to a previous study with some modifications^33^. In brief, bacteria were grown in six-well plates for 24 h at 37 °C, then plates were chilled for 1 h at 4 °C, added with 1 μL of 0.5 M EDTA per well. The supernatants were discarded, and the unwashed biofilms were resuspended in 400 μL of RB buffer (50 mM Tris-HCl, 10 mM ETDA, 500 mM NaCl, pH 8.0) and transferred into precooling tubes. After centrifugation at 18,000×*g* for 5 min at 4 °C, 200 μL of supernatant was transferred to a new chilled tube containing 300 μL of TE buffer (10 mM Tris-HCl, 1 mM EDTA, pH 8.0). Proteins were removed once with an equal volume of phenol/chloroform/isoamyl alcohol (25:24:1) and once with chloroform/isoamyl alcohol (24:1), DNA was then precipitated by adding three volumes of ice-cold 100% ethanol and 1/10 volume of 3 M sodium acetate to the aqueous phase. Precipitations were incubated overnight at −20 °C. The next day, the ethanol-precipitated DNA was collected by centrifugation at 18,000×*g* for 20 min at 4 °C, followed by washing with ice-cold 70% ethanol, air-dried, and dissolved in 20 μL of TE buffer. eDNA was quantified using NanoDrop 1000 spectrophotometer. To account for potential differences in biomass, the average OD600 values of each unwashed biofilm were determined. The nanogram of eDNA per biomass of each biofilm was then calculated via dividing its total eDNA (ng) by its corresponding OD600 value. The eDNA samples from XQ and XQW were also compared via running on 1.0% agarose gels. Experiments were repeated twice.

### RT-qPCR

The overnight cultures of XQ and XQW were transferred into fresh TSB at the dilution of 1:100, respectively. Bacteria at the later growth stages (6 h after inoculation) were harvested; the total RNA and corresponding cDNA were prepared using RNAprep Pure Cell/Bacteria Kit (TIANGEN) and RevertAid First Stand cDNA Synthesis Kit (Thermo Scientific), respectively. RT-qPCR was performed to detect the toxin genes (*RNAIII*, *seb*, *lukF*, *hla*, *hlg*, *lipA*, and *sspA*) in *S. aureus* strains of interest, and the expression level of genes related to biofilm formation, including *nuc*, *fnbA*, *fnbB*, *clfA*, and *icaA*, were also determined using primers listed in Supplementary Table S2. The relative expression levels of tested genes were normalized to that of 16S RNA gene or *gyrB*.

### Construction of the *nuc* promoter-*LacZ* reporter strain

The fragment spans −334 to +72 of *nuc* gene promoter region was amplified from *S. aureus* Newman using primer pairs listed in Supplementary Table S2, and the PCR product was inserted into pOS1 vector through *Eco*RI and *Bam*HI digestion. The recombinant plasmid was transformed into *E. coli* DH5a and verified by sequencing. Correctly constructed pOS1-*nuc* promoter-*LacZ* reporter plasmid was transformed into *S. aureus* RN4220, and then transformed into *S. aureus* Newman and Newman-SigB(Q225P), respectively.

### β-galactosidase assay

Both Newman and Newman-SigB(Q225P) carrying *nuc* promoter-*LacZ* reporter were cultured overnight and washed twice with PBS and diluted 100-fold in fresh TSB medium, respectively. Bacteria cells in 2 mL culture were harvested after being cultured for 3 h at 37 °C with shaking, resuspended in 100 μL of AB buffer (100 mM KH_2_PO_4_, 100 mM NaCl, pH 7.0)^52^. After treatment with lysostaphin (1 mg/mL, Sigma) for 15 min at 37 °C, the suspension was added with another 900 μL of ABT solution (AB buffer containing 0.1% TritonX-100). Fifty microliters of the solution were then mixed with 10 μL of 4-methylumbelliferyl-β-d-galactoside (4MUG, 4 mg/mL, Sigma) and incubated for 1 h at room temperature. Then, 20 μL of the sample was mixed with 180 μL of ABT solution, and the reaction was monitored at 445 nm with an excitation wavelength of 365 nm. All samples were tested in triplicate.

### EMSA

A DNA fragment derived from the promoter region of *nuc* gene was amplified from *S. aureus* Newman with primers listed in Supplementary Table S2. A DNA fragment from *saeR* gene coding region was also amplified and served as negative control. The PCR products were purified utilizing Wizard^®^ SV Gel and PCR Clean-Up System (Promega). SigB, as well as SigB(Q225P) proteins, were prepared in our laboratory. In brief, *sigB* and *sigB(Q225P)* genes were cloned into a pET22b vector, respectively. Corresponding proteins with a 6× His-Tag at the C-terminal were purified via Ni-NTA column and identified by SDS-PAGE and western blot. The binding reactions of EMSA were performed in a buffer system (10 mM Tris-HCl, pH 7.5, 1 mM EDTA, 50 mM NaCl, and 5% glycerol) for 30 min at room temperature. The amount of DNA fragment was maintained at 10 pmol per reaction while the amount of proteins was increased from 0 to 18 μg. Reaction mixtures were then electrophoresed on a 5% nondenaturing polyacrylamide gel at 80 V for 2–3 h at 4 °C. Gels were stained with GoldView (Solarbio) and observed under UV light. To compare the DNA binding ability of SigB and SigB(Q225P), the gray value of free probe in each lane was analyzed using ImageJ software. The gray value of free probe in first lane (0 μg protein) was adjusted to 1.0, and the relative gray values in other lanes were calculated.

### BLI assay

The BLI assay was performed to quantitatively detect the DNA binding ability of SigB and SigB(Q225P) using Octet K2 system (ForteBio). Biotin-labeled *nuc* promoter region was amplified by PCR using primer pairs (Biotin-labeled *sigB* promoter-5′ and *sigB* promoter-3′). PCR products were purified using the Wizard SV gel and PCR clean up system (Promega), quantified using a NanoDrop 1000 (Thermo), and loaded onto Read^TM^ Streptavidin (SA) biosensors (ForteBio). In a typical binding assay, SigB and BSA proteins (500 nM, dissolved in PBS, pH 7.4) were firstly used to check the specificity of the system. Then, SigB and SigB(Q225P) proteins at different concentrations (125 and 250 nM, dissolved in PBS, pH 7.4) were used to evaluate their DNA binding abilities as following: baseline step (40 s), association step (120 s), dissociation step (180 s), and regeneration step (45 s). Data were treated via Octet System Data Analysis software 8.1.

### Animal experiments

BALB/c mice were injected with *S. aureus* strains of interest for virulence evaluation. To evaluate the lethality, mice were injected with 4 × 10^7^ CFU of bacteria cells via tail vein (10 mice for each strain) and observed for up to 14 days. Survival was recorded and the survival curve was completed using GraphPad Prism 5 software. To detect the colonization ability of *S. aureus* strain of interest, mice were injected with 1 × 10^7^ CFU of bacteria cells through tail vein (10 mice for each strain). Four days later, all mice were sacrificed and the bacterial loads in liver, kidney, and liver tissues were counted after inoculation of serially diluted organ suspensions on TSB plates and cultured for 24 h at 37 °C, respectively. For skin abscess formation, mice were first treated with 6% Na_2_S to remove the back hair, and then subcutaneously injected with 1 × 10^7^ CFU of bacteria cells. Skin abscess formations were observed and recorded up to 10 days.

### Statistical analysis

Statistical analysis of results was carried out using GraphPad Prism 5. Unpaired two-tailed Student’s *t*-test was used to treat samples between two groups. The differences between multiple groups were analyzed by one-way analysis of variance (ANOVA) with Turkey’s multiple-comparison test. Each experiment was carried out at least thrice. Results are presented as mean ± standard deviations (SD), and a *P* value less than 0.05 was considered statistically significant. *P *<* 0.05, **P *<* 0.01, ***P *<* 0.001, and ns represented no significance.

## Electronic supplementary material

Supplementary information
