# Supplementary material for: A novel SigB(Q225P) mutation in Staphylococcus aureus retains virulence but promotes biofilm formation
Source: Emerg Microbes Infect. 2018 Apr 25;7:72. doi: 10.1038/s41426-018-0078-1 (PMC5915575; doi:10.1038/s41426-018-0078-1)
Supplement: Supplementary file 1 — Supplementary information [file 41426_2018_78_MOESM1_ESM.docx]

Supplementary information


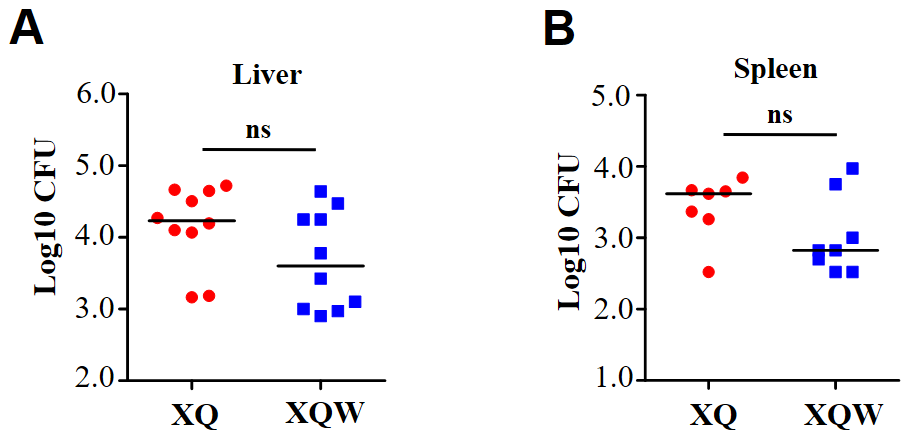


**Supplementary Figure S1** Evaluation of bacterial loads of XQ and XQW in mouse organs**.** Mice were injected with 1×10^7^ CFU of XQ and XQW strains through tail vein, respectively. Four days later, mice were sacrificed, and the bacterial load in liver (**A**) and spleen (**B**) were determined. Results were presented in scattered dot plot with a horizontal line representing the median. Unpaired *t*-test was performed, ns represents no significance.


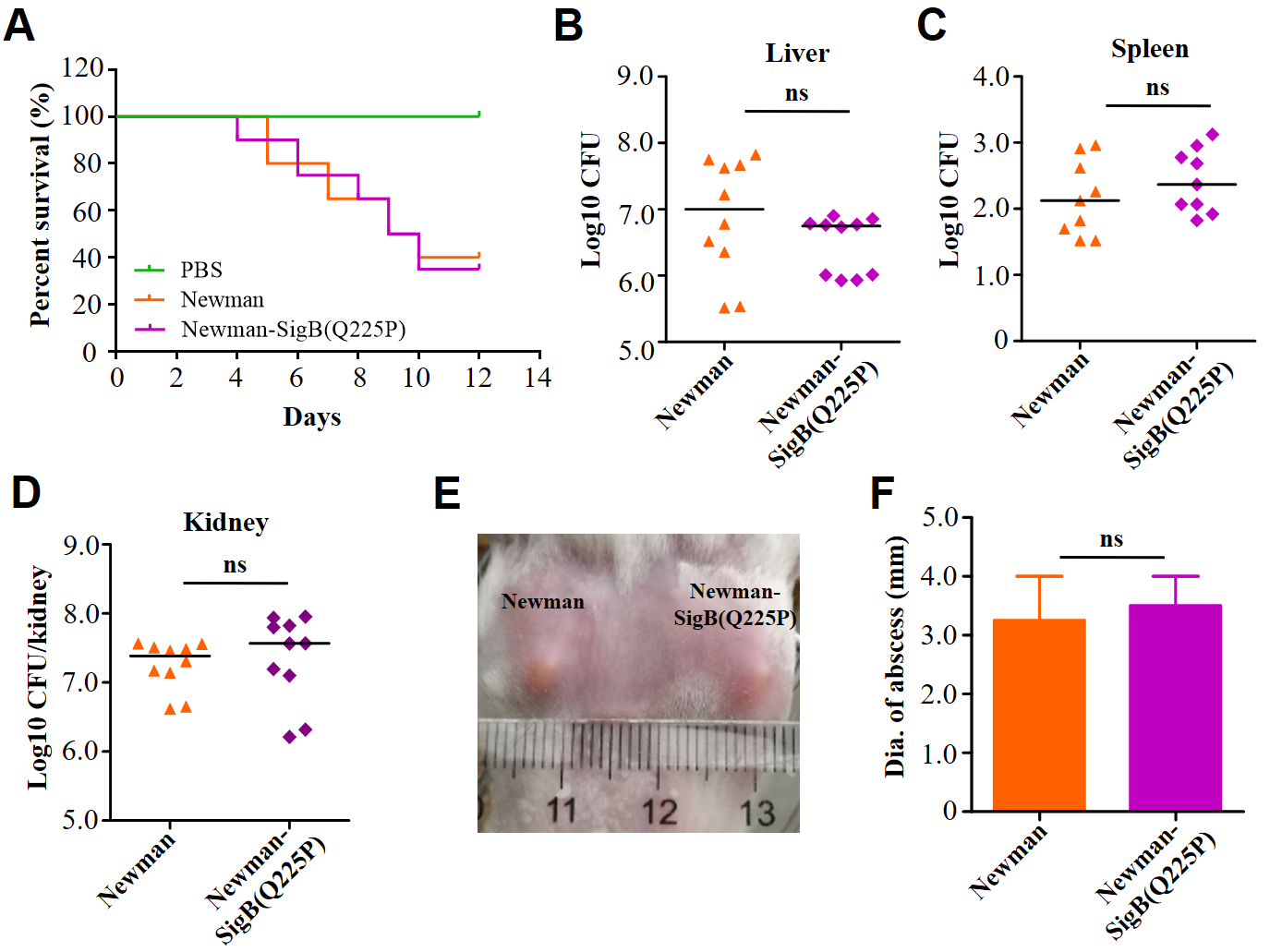


**Supplementary Figure S2** Evaluation of virulence of Newman and Newman-SigB(Q225P) in mice. **(A)** Survival analysis. Mice were injected through tail vein with 4×10^7^ CFU of Newman, Newman-SigB(Q225P), or PBS, the survival rates were calculated. The Number of mice used: *n* = 10. **(B-D)** Bacterial loads. Mice were injected through tail vein with 1×10^7^ CFU of Newman or Newman-SigB(Q225P), bacterial load in liver (**B**), spleen (**C**), and kidney (**D**) was counted four days post-injection. **(E)** Skin abscess formation. The hairs on the back of the mice were removed using 6% Na_2_S, then mice were respectively injected with Newman and Newman-SigB(Q225P) on each side. The skin abscesses were photographed 4 days post-injection. **(F)** The diameter of abscess area was measured and represented as mean ± SD (*n* ≥ 3), ns represents no significance.


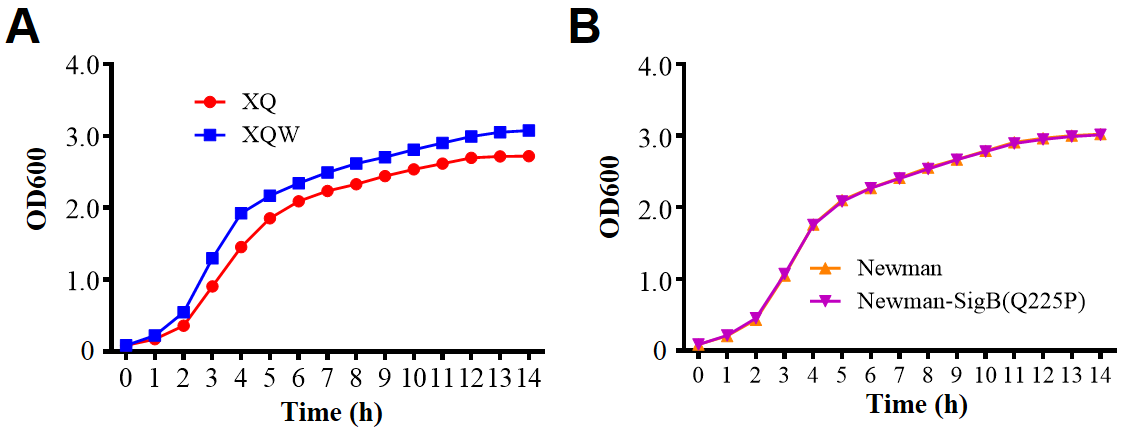


**Supplementary Figure S3** Determination of bacterial growth curves. Overnight cultured bacteria were inoculated into 50 mL of fresh TSB medium at the dilution of 1:100, and were cultured at 37 °C with shaking. One milliliter of culture was taken once every hour, and the OD600 value was detected. Experiments were performed until the bacterial growth entered into stationary phase, and were repeated thrice. The growth curves of (**A**) XQ and XQW strains, as well as (**B**) Newman and Newman-SigB(Q225P), were drawn via GraphPad Prism software 5. The generation times of these four strains were determined as previously described ^[1]^, and the results demonstrated that XQ, XQW, Newman, and Newman-SigB(Q225P) had a generation time of 54, 46.2, 50.4, and 49.9 min, respectively.


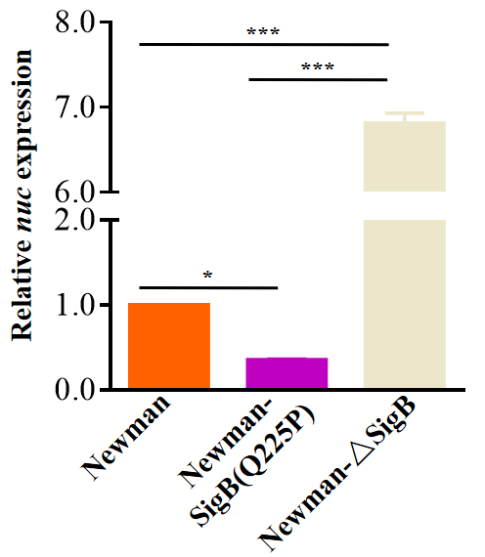


**Supplementary Figure S4** SigB(Q225P) mutation reduced *nuc* expression in Newman strain. The total RNA and corresponding cDNA were prepared. RT-qPCR was performed to detect the *nuc* gene expression in Newman, Newman-SigB(Q225P), and Newman-ΔSigB strains. The expression level of *nuc* gene was firstly normalized to that of reference *gyrB* gene, then the relative expression of *nuc* gene in Newman was set to 1.0, and the relative expression of *nuc* gene in Newman derivatives were calculated and indicated. Results were shown by mean ± SD (*n* = 3). One-way ANOVA was used to analyze the significant difference between groups. * *P* < 0.05, *** *P* < 0.001.


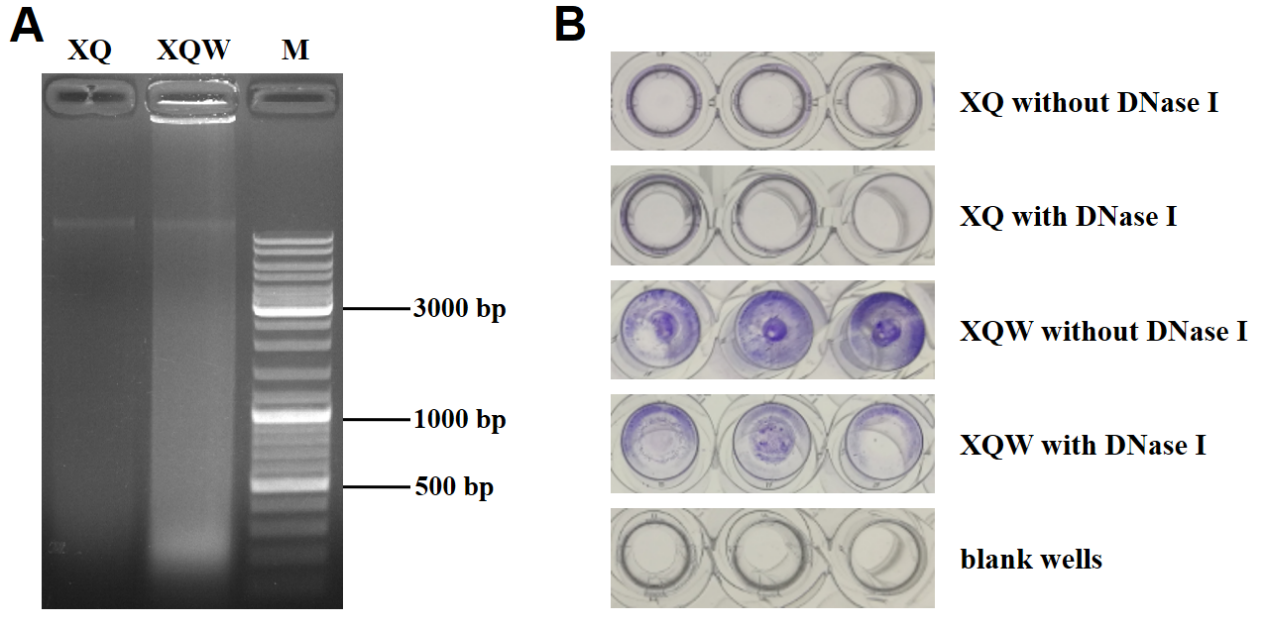


**Supplementary Figure S5** Detection of eDNA in XQ and XQW derived biofilms. **(A)** eDNA derived from XQ and XQW biofilms were extracted and were run with 1% agarose gels and photographed. **(B)** Biofilm formation of XQ and XQW cultured in 96-well plates for 24 h with or without the addition of DNase I (1U/well). Wells were washed thrice with water, stained with 0.1% crystal violet for 1 min, washed thrice again, air dried, and photographed.


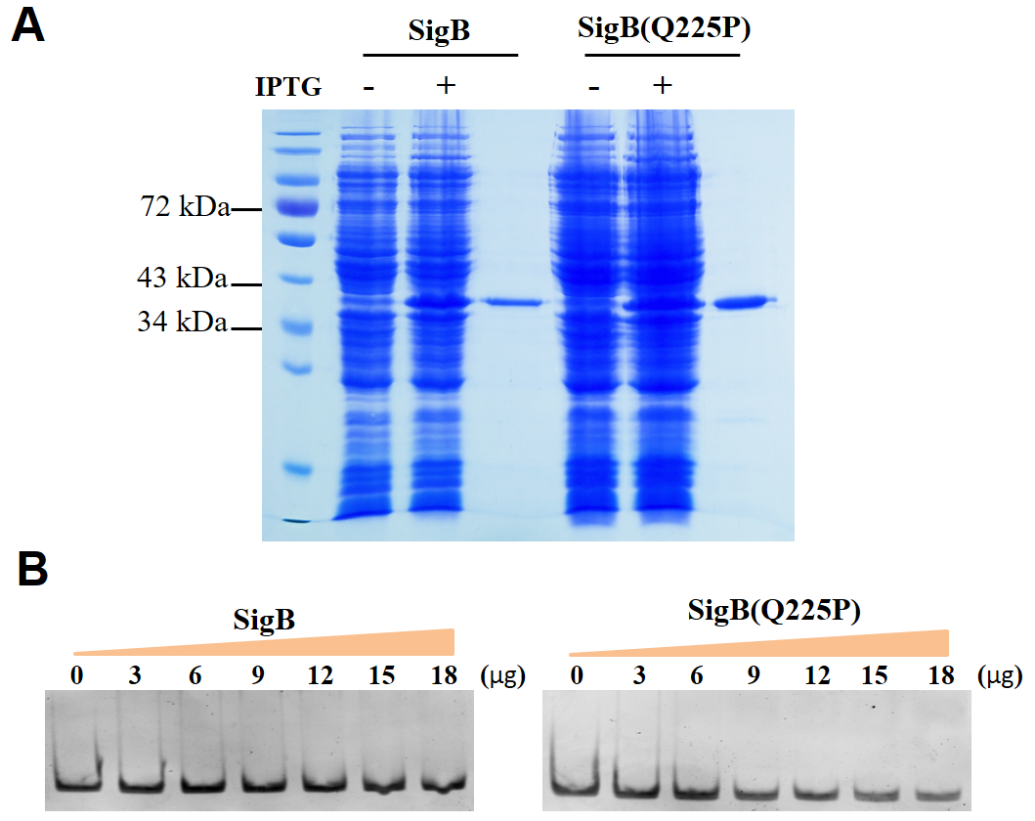


**Supplementary Figure S6** (**A**) SigB and SigB(Q225P) proteins were purified and evaluated via SDS-PAGE. (**B**) The interaction between unrelated DNA fragments (amplified from *saeR* coding region) and SigB or SigB(Q225P) proteins was detected.

**Supplementary Table S1** Mutations in *S. aureus* XQW genome compared with XQ

| Gene name | Gene encoding product | Information of site mutation |
| --- | --- | --- |
| ASU36_00065 | serine O-acetyltransferase | 14807, base A deletion. |
| ASU36_00075 | ribonuclease III | 16841, base C to A;  16846, base G to A;  16849, base T insertion;  16850, base G to T;  16852, base C to T;  16853, base G to T;  16859, base G to T. |
| ASU36_00225 | hydrolase | 51138, base T to C;  51561, base T to C;  51567, base T to C;  51615, base T to C;  51621, base T to C;  51628, base T to A;  51629, base C to G. |
| ASU36_00390 | transposase | 82307, base G to C;  82308, base G to A;  82311, base T insertion;  82314, base C to T;  82315, base G to A;  82319, base A deletion;  82320, base C to T;  82321, base G to T. |
| ASU36_00395 | hypothetical protein | 84045, base A insertion. |
| ASU36_00470 | ABC transporter substrate-binding protein | 97420, base A deletion. |
| ASU36_00480 | haloacid dehalogenase | 99384, base C to T;  99385, base A to G;  99389, base A to T;  99394, base T deletion;  99395, base G to A;  99398, base A to T;  99399, base A to C;  99401, base A to G. |
| ASU36_00615 | glycosyl transferase | 124716, base C deletion. |
| ASU36_00635 | nucleoside permease | 130500, base G to A;  130503, base G to A;  130506, base T to A;  130508, base A insertion;  130510, base T to A;  130515, base T insertion;  130516, base C to T. |
| ASU36_00720 | DNA-binding response regulator | 145551, base A deletion. |
| ASU36_00760 | AraC family transcriptional regulator | 155288, base C deletion;  155292, base T to C;  155293, base A to T;  155294, base T to C;  155295, base T to A. |
| ASU36_00885 | hypothetical protein | 177275, base A insertion. |
| ASU36_01040 | heme ABC transporter ATP-binding  protein | 206843, base A insertion;  206844, base T to G;  206845, base A to T;  206850, base C to G;  206852, base G insertion;  206855, base T to G;  206858, base C to A. |
| ASU36_01080 | 7-cyano-7-deazaguanine reductase | 217440, base T insertion. |
| ASU36_01240 | hydrolase | 250045, base A deletion. |
| ASU36_01290 | RNase adaptor protein RapZ | 261741, base A to C;  261745, base T to C;  261748, base G insertion;  261757, base A to C. |
| ASU36_01300 | hypothetical protein | 263990, base A insertion. |
| ASU36_01325 | DUF4887 domain-containing protein | 269928, base T insertion;  269930, base T to A;  269932, base A insertion;  269934, base G to A;  269935, base T insertion;  269938, base A to G;  269940, base A to G;  269941, base A to C;  269944, base A to C;  269946, base C to G. |
| ASU36_01395 | hypothetical protein | 285459, base A deletion. |
| ASU36_01465 | hypothetical protein | 298195, base T insertion. |
| ASU36_01710 | hypothetical protein | 336992, base A deletion. |
| ASU36_01755 | Na+/H+ antiporter subunit D | 345204, base A deletion. |
| ASU36_01875 | O-acetyltransferase | 378610, base A to T. |
| ASU36_01890 | hypothetical protein | 382835, base A insertion. |
| ASU36_01960 | hypothetical protein | 396607, base A insertion;  396608, base G to A. |
| ASU36_01970 | peptide ABC transporter substrate binding protein | 399028, base A insertion. |
| ASU36_01985 | hypothetical protein | 402208, base T deletion. |
| ASU36_02055 | enoyl-ACP reductase | 416674, base T deletion;  416675, base T deletion. |
| ASU36_02170 | bacteriocin transporter | 439570, base A deletion. |
| ASU36_02240 | 2-succoinyl-5-enolpyruvyl-6-hydroxy-3-cyclohexene-1-carboxylate synthase | 451462, base T to C;  451474, base T to A. |
| ASU36_02325 | hypothetical protein | 470535, base C to T;  470537, base G to A;  470541, base A to C;  470543, base T to A;  470544, base C insertion;  470546, base T insertion;  470548, base C to A;  470550, base G to A;  470551, base C to T. |
| ASU36_02355 | 5-(carboxyamino) imidazole ribonucleotide synthase | 477139, base G to A. |
| ASU36_02400 | phosphoribosylamine-glycine ligase | 486770, base G to A;  486771, base C to A;  486775, base G deletion;  486777, base T to G;  486778, base T to A;  486779, base T to G;  486782, base A to G;  486783, base T to C;  486784, base G to T;  486788, base A to G. |
| ASU36_02600 | pyruvate carboxylase | 528341, base A deletion. |
| ASU36_02605 | hypothetical protein | 529768, base T insertion. |
| ASU36_02635 | hypothetical protein | 533989, base C insertion. |
| ASU36_02680 | heme uptake protein IsdB | 539508, base T insertion;  539510, base A insertion;  539515, base A deletion;  539516, base C deletion;  539518, base A deletion;  539521, base A deletion;  539524, base T to C;  539527, base C insertion. |
| ASU36_02720 | hypothetical protein | 547019, base T deletion. |
| ASU36_02740 | ribonuclease H III | 552337, base T deletion. |
| ASU36_02760 | endonuclease MutS2 | 558401, base A deletion. |
| ASU36_03250 | beta-ketoacyl-ACP reductase | 649753, base A to C;  649754, base G to C;  649757, base G to T;  649760, base G deletion;  649761 , base T to A;  649762, base T to C;  649764, base A to C;  649766, base G to C;  649770, base T to A;  649771, base G to A. |
| ASU36_03270 | chromosome segregation protein SMC | 654501, base C insertion;  654502, base T to C;  654505, base A to T;  654506, base A to T;  654509, base A to G;  654511, base C deletion;  654517, base A to C;  654519, base T to A. |
| ASU36_03335 | succinyl-CoA ligase subunit alpha | 668184, base A to T;  668185, base G to A;  668188, base G deletion;  668189, base T to A;  668190, base G to A;  668194, base T to C;  668195, base G to T. |
| ASU36_03350 | DNA processing protein DprA | 670273, base G deletion. |
| ASU36_03415 | isoprenyl transferase | 682890, base A to T;  682895, base T to C;  682896, base A to C;  682898, base T deletion;  682900, base A to C;  682904, base T to A;  682907, base A to G;  682908, base G to C |
| ASU36_03475 | bifunctional riboflavin kinase/FMN | 698856, base T to C;  698857, base T insertion;  698858, base C insertion;  698859, base G insertion;  698863, base T deletion;  698868, base C to T;  698872, base A to T. |
| ASU36_03485 | polyribonucleotide nucleotidyltranferase | 701861, base T deletion. |
| ASU36_03500 | transcriptional regulator | 707493, base A to G;  707494, base C to A;  707495, base G to A;  707498, base C to T;  707499, base A deletion;  707500, base G to T;  707501, base C to A;  707503, base G to A;  707505, base T to A;  707507, base G to T;  707509, base G to T;  707510, base A to G. |
| ASU36_03510 | hypothetical protein | 710121, base A deletion. |
| ASU36_03555 | metallophosphoesterase | 718837, base G to A;  718838, base G to C;  718839, base C to T;  718845, base T to A;  718847, base A insertion;  718848, base C to T;  718850, base G to A;  718851, base C to G;  718855, base T to A. |
| ASU36_03565 | 2-oxoacid ferredoxin oxidoreductase subunit beta | 721388, base A deletion. |
| ASU36_04035 | guanosine monophosphate reductase | 808364, base A to C;  808365, base T to C;  808371, base C insertion;  808372, base C to T;  808377, base A to G;  808381, base A to T. |
| *gyrB* | DNA topoisomerase IV subunit B | 827674, base T insertion. |
| ASU36_04180 | DNA repair protein | 840867, base A insertion. |
| ASU36_04425 | hypothetical protein | 890077, base A deletion. |
| ASU36_04690 | hypothetical protein | 972949, base G to T;  972951, base T to C;  972952, base T to A;  972954, base T to A;  972955, base T deletion;  972957, base A deletion;  972958, base G to A;  972962, base T insertion. |
| ASU36_04755 | hypothetical protein | 985141, base T insertion. |
| ASU36_04765 | hypothetical protein | 987990, base T insertion. |
| ASU36_04830 | hypothetical protein | 997246, base T to G;  997275, base T to C;  997758, base T to G;  997759, base C to T;  997771, base A to G;  997797, base C to T. |
| ASU36_04850 | LeucotoxinLukD | 999340, base G insertion. |
| ASU36_04870 | phage protein | 1003748, base T insertion. |
| ASU36_04885 | hypothetical protein | 1005412, base T deletion;  1005779, base A insertion. |
| ASU36_04890 | hypothetical protein | 1007236, base G insertion. |
| ASU36_04910 | phage tail protein | 1013008, base T insertion. |
| ASU36_04975 | terminase | 1025204, base T insertion. |
| ASU36_05035 | hypothetical protein | 1031964, base A deletion. |
| ASU36_05100 | hypothetical protein | 1038777, base T insertion. |
| ASU36_05140 | transcriptional regulator | 1041347, base A insertion. |
| ASU36_05160 | hypothetical protein | 1043426, base C to T. |
| ASU36_05175 | hypothetical protein | 1046836, base C deletion. |
| ASU36_05200 | SMC-Scp complex subunit ScpB | 1052068, base A to G;  1005470, base C to A;  1005476, base C to A;  1005477, base G deletion;  1005481, base A to T;  1005484, base A to G;  1005486, base T to A. |
| ASU36_05265 | hypothetical protein | 1064212, base T, insertion. |
| ASU36_05480 | MFS transporter | 1099959, base A insertion. |
| ASU36_05495 | hypothetical protein | 1103831, base A deletion. |
| *sigA* | RNA polymerase sigma factor SigA | 1112326, base T insertion. |
| ASU36_05545 | DNA primase | 1113599, base A insertion. |
| ASU36_05630 | molecular chaperone DnaJ | 1128290, base A insertion. |
| ASU36_05645 | hypothetical protein | 1132870, base C deletion. |
| ASU36_05920 | hypothetical protein | 1186837, base T insertion. |
| ASU36_05940 | Holliday junction DNA helicase RuvB | 1192598, base G insertion. |
| ASU36_05980 | hypothetical protein | 1198323, base T deletion. |
| ASU36_05990 | hypothetical protein | 1199300, base A insertion. |
| ASU36_06020 | hypothetical protein | 1205314, base A deletion. |
| ASU36_06120 | gamma-aminobutyrate permease | 1221645, base G insertion. |
| ASU36_06140 | helicase DnaB | 1226527, base T deletion. |
| ASU36_06150 | glyceraldeyde-3-phosphate dehydrogenase | 1229051, base T insertion. |
| ASU36_06175 | sensor histidine kinase | 1237289, base C to G;  1005491, base T to A;  1237292, base T to C;  1237293, base G to A;  1237297, base C deletion;  1237300, base A to G;  1237302, base A to C; |
| ASU36_06370 | tyrosline-tRNA ligase | 1280743, base A insertion. |
| ASU36_06385 | hypothetical protein | 1284266, base A to T;  1284267, base T to A;  1284271, base C to T;  1284272, base C deletion;  1284273, base C insertion;  1284275, base G to A;  1284278, base G to A;  1284281, base T to C;  1284282, base G to A. |
| ASU36_06435 | hypothetical protein | 1295297, base T insertion. |
| ASU36_06520 | hypothetical protein | 1315502, base T insertion. |
| ASU36_06615 | autolysin | 1341256, base A insertion. |
| ASU36_06620 | hypothetical protein | 1342163, base A insertion. |
| ASU36_06645 | hypothetical protein | 1363016, base A to T;  1363017, base G to A;  1363019, base A to T;  1363021, base A insertion;  1363024, base T to A. |
| ASU36_06855 | hypothetical protein | 1381852, base A insertion. |
| ASU36_07675 | antibiotic ABC transporter ATP-binding protein | 1515555, base T insertion. |
| ASU36_07795 | phage tail tape measure protein | 1538611, base C to A;  1538613, base C to T;  1538614, base C to T;  1538616, base G to T;  1538617, base T to G;  1538619, base T insertion;  1538620, base C to T;  1538625, base G to A;  1538629, base T to A. |
| ASU36_08065 | hypothetical protein | 1564736, base A deletion. |
| ASU36_08070 | site-specific integrase | 1566033, base A deletion. |
| ASU36_08100 | hypothetical protein | 1572776, base T insertion. |
| ASU36_08235 | hypothetical protein | 1599620, base G deletion;  1599674, base G deletion. |
| ASU36_08240 | hypothetical protein | 1600162, base T deletion;  1600698, base C insertion. |
| ASU36_08305 | L-threonine dehydratase biosynthetic IIvA | 1613130, base A insertion. |
| *sigB* | RNA polymerase sigma factor SigB | 1623123, base T to G. |
| ASU36_08305 | membrane protein | 1628451, base A to C;  1628452, base T to A;  1628454, base A to C;  1628459, base C to T;  1628461, base G insertion;  1628463, base T to A;  1628469, base C to T;  1628470,base A to T |
| ASU36_08445 | hypothetical protein | 1641730, base T insertion. |
| ASU36_08645 | transcription termination factor Rho | 1674841, base T insertion. |
| ASU36_08905 | tRNA-Tyr | 1730417, base G insertion. |
| ASU36_09115 | hypothetical protein | 1758094, base T insertion. |
| ASU36_09215 | hypothetical protein | 1776368, base C insertion;  1776372, base T to A;  1776380, base C to A;  1776381, base T to A;  1776382, base G to C;  1776384, base T to A;  1776385, base T to C;  1776403, base A to T. |
| ASU36_09315 | 30S ribosomal protein S13 | 1793834, base A insertion. |
| *rpsN* | RpsN | 1799366, base T deletion. |
| ASU36_09430 | 50S ribosomal protein L4 | 1804234, base T insertion. |
| ASU36_09455 | DNA topoisomerase III | 1809213, base A deletion. |
| ASU36_09500 | membrane protein | 1819922, base A to G;  1819923, base T to C;  1819926, base T to C;  1819927, base G deletion;  1819928, base T to G;  1819929, base C to A;  1819934, base T to A;  1819935, base T to A;  1819936, base T to G;  1819937, base T to C. |
| ASU36_09530 | hypothetical protein | 1823661, base C deletion. |
| *ureB* | UreB | 1840443, base A to G;  1840444, base A to G;  1840445, base C to T;  1840446, base C to A;  1840449, base T deletion;  1840450, base C deletion;  1840452, base A to T;  1840456, base C to G;  1840458, base A to G;  1840461, base G to T. |
| *ureF* | urease accessory protein UreF | 1843028, base A insertion;  1840461, base G to T. |
| ASU36_09720 | hypothetical protein | 1855205, base A deletion. |
| ASU36_10045 | L-lactate permease | 1919818, base A insertion. |
| ASU36_10050 | hypothetical protein | 1921707, base T deletion. |
| ASU36_10110 | PTS maltose transporter subunit II BC | 1931106, base T deletion. |
| ASU36_10435 | hypothetical protein | 1995300, base A insertion. |
| ASU36_10450 | hypothetical protein | 1999494, base C insertion. |
| ASU36_10460 | hypothetical protein | 2001507, base A insertion. |
| ASU36_10560 | hypothetical protein | 2021292, base A insertion. |
| ASU36_10685 | hypothetical protein | 2044315, base T insertion. |
| ASU36_10740 | hypothetical protein | 2055823, base A to T;  2055824, base G deletion;  2055840, base A to G;  2059360, base A to G;  2059437, base A deletion;  2059485, base C insertion. |
| ASU36_11010 | ferrous iron transport protein B | 2114968, base T insertion. |
| ASU36_11165 | short –chain dehydrogenase | 2145478, base C insertion. |
| ASU36_11520 | hypothetical protein | 2213553, base A deletion. |
| ASU36_11540 | phage infection protein | 2219086, base T insertion. |
| ASU36_11690 | hypothetical protein | 2260628, base C deletion;  2260682, base C deletion. |
| ASU36_11725 | hypothetical protein | 2266362, base T insertion. |
| ASU36_11790 | adhesin | 2278149, base G to A;  2278179, base T to C. |
| *dnaA* | chromosomal replication initiation protein DnaA | 2301796, base G insertion. |
| ASU36_12065 | membrane protein | 2341897, base T insertion. |
| ASU36_12075 | hypothetical protein | 2342813, base T deletion. |
| ASU36_12160 | oleate hydratase | 2363007, base T insertion. |
| *sbnG* | siderophore biosynthesis protein SbnG | 2384428, base C insertion. |
| ASU36_12320 | purine-nucleoside phosphorylase | 2397708, base A to T. |
| ASU36_12485 | aldehyde dehydrogenase | 2434035, base C insertion. |
| ASU36_12635 | hypothetical protein | 2476329, base T insertion. |
| ASU36_12755 | coagulase | 2507306, base A deletion. |
| ASU36_12795 | hypothetical protein | 2519498, base T deletion. |
| ASU36_12820 | L-lactate dehydrogenase | 2523515, base T insertion. |
| ASU36_12935 | DNA binding response regulator | 2547755, base A insertion. |
| ASU36_12950 | hypothetical protein | 2552012, base A deletion. |
| ASU36_12980 | hypothetical protein | 2557497, base C deletion. |
| *esaA* | protein EsaA | 2572524, base T to C; |
| ASU36_13110 | hypothetical protein | 2585315, base G to A;  2585331, base G deletion. |
| ASU36_13140 | hypothetical protein | 2589190, base A deletion;  2589443, base T to C;  2589449, base A to T. |
| ASU36_13230 | hypothetical protein | 2608155, base C deletion;  2608852, base A insertion. |
| ASU36_13495 | DNA-binding protein | 2659211, base T insertion. |
| ASU36_13630 | hypothetical protein | 2676534, base T insertion. |
| ASU36_13810 | hypothetical protein | 2709259, base T deletion. |
| ASU36_13820 | restriction endonuclease subunit S | 2711880, base A to G;  2711883, base A to G;  2711896, base G to A. |
| ASU36_13850 | hypothetical protein | 2717782, base A insertion. |
| ASU36_14160 | stage V sporulation protein G | 2779563, base T insertion. |
| ASU36_14265 | lysine-tRNA ligase | 2800832, base T insertion. |

**Supplementary Table S2** Primers used in this study

| Primers | Sequence (5'→3') | Notes/Reference |
| --- | --- | --- |
| **PCR** |  |  |
| up-*sigB*-5′ | CCGGAATTCCTTCAACACGTTGTAATTTG | *sigB* gene upstream amplification |
| up-*sigB*-3′ | CGGGGTACCAATTTGTTTATTAATGATACGT |  |
| *sigB*(Q225P)-5′ | GGGGTACCCTATTTATGTGCTGCTTCTT | *sigB*(Q225P) allelic replacement |
| *sigB*(Q225P)-3′ | GCTCTAGATCACCTGAGCAAATTAACCA |  |
| down-*sigB*-5′ | CGGGGTACCTTAGCTGATTTCGACTCTTT | *sigB* gene downstream amplification |
| down-*sigB*-3′ | CCCAAGCTTCAAGATAAATTTTACGAAGTTA |  |
| *sigB*promoter-5′ | CGGAATTCGATGATATGACTATTTTG | *sigB* gene promoter amplification |
| *sigB*promoter-3′ | GGGGTACCCATTTCATTACACTCCTACT |  |
| *sigB*-C-5′ | GGGGTACCATGGCGAAAGAGTCGAAATC | *sigB* cds amplification |
| *sigB*-C-3′ | CCAAGCTTTCAGTGGTGGTGGTGGTGGTGT  TGATGTGCTGCTTCTTGTA |  |
| *sigB*-exp-5’ | CGGAATTCGGCGAAAGAGTCGAAATCAG | Expression of SigB/SigB(Q225P) |
| *sigB*-exp-3’ | CCCAAGCTTTTGATGTGCTGCTTCTTGTA |  |
| *nuc* promoter-5’ | CGGAATTCGTAAATTATAAGTTATACATCTCG | *nuc* gene promoter amplification |
| *nuc* promoter-3’ | CGGGATC CGATAGCCATCCCTATAAGTA |  |
| *nuc* probe-5’ | CCAAAGTAAATTATAAGTTATACATCTCG | EMSA |
| *nuc* probe-3’ | CACCTCTTTCTTTTTAGTTAATTTTAATAT |  |
| *saeR*-5’ | CGCCTTAACTTTAGGTGCAGATGAC | EMSA (negative control) |
| *saeR*-3’ | ACGCATAGGGACTTCGTGACCATT |  |
| **RT-qPCR** |  |  |
| *gyrB*-5’ | CAAAGACCTCCCAATGTT | this study |
| *gyrB*-3’ | CGGCATCAGTCATAATGACGAT |  |
| *nuc*-5’ | AATATGGACGTGGCTTAGCGT | this study |
| *nuc*-3’ | TTGACCTGAATCAGCGTTGTCTT |  |
| *fnbA*-5’ | CCAGGTGGTGGTCAGGTTAC | [2] |
| *fnbA*-3’ | TGTGCTTGACCATGCTCTTC |  |
| *fnbB*-5’ | ACCTGCTAAAGAAGAACC | [2] |
| *fnbB*-3’ | CGTAATAACGCTAAACCTA |  |
| *clfA*-5’ | TTTCAACAACGCAAGATA | [2] |
| *clfA*-3’ | GCTACTGCCGCTAAACTA |  |
| *icaA*-5’ | TACTATTTCGGGTGTCTTCA | [2] |
| *icaA*-3’ | CAAAGACCTCCCAATGTT |  |
| *RNAIII*-5’ | CATGGTTATTAAGTTGGGATGGC | this study |
| *RNAIII*-3’ | GAAGGAGTGATTTCAATGGCACA |  |
| *hla*-5’ | AAGTGGTTTAGCCTGGCCTTCA | this study |
| *hla*-3’ | TCGAAACATTTGCACCAATAAGG |  |
| *seb*-5’ | TGTATGTATGGTGGTGTAACTGAGC | this study |
| *seb*-3’ | AGGCGAGTTGTTAAATTCATAGAGT |  |
| *lukF*-5’ | TGTGCTTCAACTTTCCAACCTACA | this study |
| *lukF*-3’ | CCAAAAAATCAGGATGAATCAAGAG |  |
| *lipA*-5’ | ACACAATGTTAGGGTTCAACGACG | this study |
| *lipA*-3’ | AAGAGTAGACTTCGGGTTGGCTC |  |
| *hlg*-5’ | TTCGCTTGTATCGCTTGAACCT | this study |
| *hlg*-3’ | TCATTCGCCACTGAATCAGGTC |  |
| *sspA*-5’ | CTACAACTACACCGGAAGCAATAAA | this study |
| *sspA*-3’ | ACAGACAAACAGCAAACACCTAAGA |  |

**References**

1. [Aiba Y](https://www.ncbi.nlm.nih.gov/pubmed/?term=Aiba%20Y%5BAuthor%5D&cauthor=true&cauthor_uid=23877693), [Katayama Y](https://www.ncbi.nlm.nih.gov/pubmed/?term=Katayama%20Y%5BAuthor%5D&cauthor=true&cauthor_uid=23877693), [Hishinuma T](https://www.ncbi.nlm.nih.gov/pubmed/?term=Hishinuma%20T%5BAuthor%5D&cauthor=true&cauthor_uid=23877693) *et al.* Mutation of RNA Polymerase β-Subunit Gene Promotes Heterogeneous-to-Homogeneous Conversion of β-Lactam Resistance in Methicillin-Resistant *Staphylococcus aureus.* [*Antimicrob Agents Chemother*](https://www.ncbi.nlm.nih.gov/pubmed/?term=Mutation+of+RNA+Polymerase+%01-Subunit+Gene+Promotes+Heterogeneous-to-Homogeneous+Conversion+of+%01-Lactam+Resistance+in+Methicillin-Resistant+Staphylococcus+aureus) **57,** 4861–4871 (2013).
2. [Kulkarni R](https://www.ncbi.nlm.nih.gov/pubmed/?term=Kulkarni%20R%5BAuthor%5D&cauthor=true&cauthor_uid=22890993), [Antala S](https://www.ncbi.nlm.nih.gov/pubmed/?term=Antala%20S%5BAuthor%5D&cauthor=true&cauthor_uid=22890993), [Wang A](https://www.ncbi.nlm.nih.gov/pubmed/?term=Wang%20A%5BAuthor%5D&cauthor=true&cauthor_uid=22890993) *et al.* Cigarette smoke increases *Staphylococcus aureus* biofilm formation via oxidative stress. *Infect Immun* **80,** 3804–3811 (2012).
